# Informed consent checklists for midurethral slings: a common-sense approach

**DOI:** 10.1007/s00192-017-3456-7

**Published:** 2017-08-29

**Authors:** G. Alessandro Digesu, Steven Swift, Victoria Handley

**Affiliations:** 1 Department of Urogynaecology, Academic Department of Obstetrics and Gynaecology, St. Mary’s Hospital, Mint Wing, South Wharf, London, W2 1NY UK; 2 Department of Obstetrics and Gynaecology, Medical University of South Carolina, Charleston, SC USA; 3 Handley Law Limited, Liverpool, England

**Keywords:** Midurethral sling, Mesh, Medical negligence, Consent, Checklist, Lawsuits

## Abstract

**Introduction and Hypothesis:**

Following the US Food and Drug Administration’s (FDA’s) warning about the use of transvaginal mesh to treat pelvic organ prolapse (POP) and the use of single-incision slings to treat incontinence, the number of lawsuits for medical negligence regarding the use of any polypropylene mesh in the vagina has increased tremendously.

**Methods:**

This same FDA document did not question the use of polypropylene midurethral slings and polypropylene for sacrocolpopexies. Surprisingly, despite all the evidence and recommendations from respected international scientific societies, we are constantly being called upon by our patients to defend the use of midurethral slings. The most common reasons for the new rash of medicolegal proceedings involving midurethral slings has to do with “breach of duties” resulting from undisclosed postoperative complications on the consent form and/or the lack of information in the medical records confirming that all possible alternative treatment options were presented to and discussed with the patient.

**Results:**

One response to these lawsuits involves the addition of preoperative checklists when performing informed consent with patients electing surgical correction of stress urinary incontinence (SUI).

**Conclusions:**

This clinical opinion provides an expert clinician’s perspectives and legal point of view on this controversial topic and discusses the role of a preoperative checklist supplementary to the standard informed consent form.

## What should clinicians do to avoid lawsuits? The experts’ point of view

### Lessons from the never-sued

With the recent US Food and Drug Administration’s (FDA’s) warning and concerns over complications from transvaginal mesh kits for prolapse and single-incision slings for incontinence has come a new-found, unreasonable amount of adverse attention to the current gold standard for treating stress urinary incontinence (SUI), namely, the polypropylene midurethral sling [1, 2]. Most, if not all, major international societies devoted to treating SUI, including the International Urogynecology Association (IUGA), the American Urogynecologic Society (AUGS), the American Urologic Association (AUA), the Society for Urodynamics and Female Pelvic Medicine and Urogenital Reconstruction (SUFU), the Royal Australian and New Zealand College of Obstetricians and Gynaecologists (RANZOG), the European Association of Urology (EAU), the American Congress of Obstetrics and Gynaecology (ACOG), and the Scientific Committee on Emerging and Newly Identified Health Risks (SCENIHR), have all issued statements supporting the use of midurethral slings as the preferred first-line surgical management for SUI [3–7]. They all make this claim because an overwhelming amount of evidence from many prospective trials (with follow-up of > 17 years in one instance) have demonstrated that the midurethral sling has equal or improved efficacy over any other surgical procedure used to treat SUI [8–14] and, by all measures, are safer and with fewer complications than any other surgical procedure to date [8–14]. Mesh-specific complication rates to treat SUI also remain low, with rates of tape erosion/extrusion around 2% and postoperative groin pain 1.3–6.4%, with most reports noting resolution of groin pain within weeks of surgery. Sexual function or dyspareunia are often a central complaint in legal issues but improved overall postoperatively, most commonly secondary to resolution of coital incontinence issues [9]. Nevertheless, despite all the evidence and recommendations from respected societies we are still constantly being called upon by our patients, the media and/or our friends in the legal community to defend the use of midurethral slings (http://www.iuga.org/default.asp?page=mus).

Undisclosed postoperative complications at the time of preoperative counselling and/or lack of information about the possible alternative therapies and/or surgery are the main reasons for medicolegal calls for breach of duty and litigation. Therefore, the debatable question between clinicians and lawyers, expert witnesses and barristers, defendants and plaintiffs is whether it is clinically appropriate or sensible to inform patients during the informed consent process? Should we discuss ad nauseam all the possible rare complications associated with any kind of surgery (including death or permanent disabilities) at the time of counselling? Should clinicians with expertise and experience in managing SUI guide, suggest, and/or decide what would be the best option, or should the patient decide which treatment to have or what to do on the basis of the information provided by her clinician?

On the one hand, if we start counselling our patients about all the rare complications from any surgery, including; permanent disabilities, untreatable chronic pain, sexual dysfunction, loss of ability to have sexual intercourse, or even death—all of which are potential with any kind of surgery—we might end up not treating any patients, leaving them to deal with their urinary incontinence (UI). Even if patients decide to proceed with surgery, this strategy will extend counselling sessions to possibly hours, providing an overwhelming amount of information that will only confuse our patients. Another concern is what should a prudent clinician record on the consent form or in the clinical notes to be sure that he/she is not liable of breach of duty or exposed to medical malpractice litigation? This is the dilemma that many clinicians face on a daily basis since the FDA warnings, which has increased litigation frenzy over polypropylene mesh implanted in the vagina [1, 2]. To help understand the legal issues surrounding informed consent, it is important to understand how we got to the current understanding by the courts regarding the patient’s role in informed consent.

### Patients have a role to play in making decisions about treatment or care

In 1957, Mr. Bolam, a voluntary patient at Friern Hospital, a mental health institution in UK, agreed to undergo electroconvulsive therapy. He was not given muscle relaxants and his body was not restrained during the procedure. He flailed about violently before the procedure was stopped, and he suffered some serious injuries, including fractures of the acetabula. Bolam sued the hospital for compensation, arguing that doctors were negligent for: (1) not issuing relaxants; (2) not restraining him; (3) not warning him about the risks involved. The expert witnesses confirmed that the majority of medical opinion was opposed to the use of relaxant drugs and manual restraints, as these could sometimes increase the risk of fracture. The judge also noted that it was common practice of the profession to not warn patients of the risk of treatment (when it is small) unless they were asked. The verdict noted that what was deemed common practice in a particular profession was highly relevant to the standard of care required. Therefore, if a doctor acts in accordance with a responsible body of medical opinion, he or she will not be deemed negligent. In other terms, there is no breach of standard of care if a responsible body of similar professionals supports the practice that caused the injury, even if the practice was not the standard of care. The ruling meant that an accused doctor need only to find experts who would testify to having done the same thing or practiced under the same standard of care as a means of establishing when a physician had or had not met their duty to the patient in providing treatment.

Since then in England, the Bolam test has been widely used in tort law to assess clinical negligence and determine the standard of care owed by professionals, such as doctors, to those whom they serve, such as patients. In 2015, an appeal court [Montgomery v Lanarkshire Health Board (Scotland) Hilary Term (2015) UKSC 11] sounded the death knell for the Bolam test in consent cases. It was concluded that the Bolam test was not appropriate in instances of consent. In this case, Montgomery (the claimant) suffered from insulin-dependent diabetes mellitus and delivered an infant via spontaneous vaginal delivery and with severe disabilities from a shoulder dystocia. It was agreed between the parties that the risk of shoulder dystocia occurring during vaginal delivery was 9–10% in the case of diabetic mothers. However, Montgomery was not told of the risk of shoulder dystocia, as, in the doctor’s opinion, the possibility of it causing a serious problem for the baby was very small. The doctor also suggested that advising of the risk would lead to most women electing for a caesarean section, something that the doctor did not agree with. On 1 October 1999, Montgomery gave birth to a baby boy at Bellshill Maternity Hospital, Lanarkshire. After his birth, as a result of complications during the delivery (shoulder dystocia), the baby was born with severe disabilities (dyskinetic cerebral palsy). It was the claimant’s case that if she was informed of the risk of shoulder dystocia, she would have elected for a caesarean section. In the proceedings, Montgomery sought damages on behalf of her son for the injuries he sustained, attributing those injuries to clinical negligence. Montgomery sued the hospital for clinical negligence on the basis of lack of information about the risk of shoulder dystocia and the fact that she was not given the option to deliver by elective caesarean section. The clinical negligence was rejected based on expert evidence of medical practice, following the approach laid down by the majority (Bolam test).

On 11 March 2015, the Supreme Court felt that the decision was unsatisfactory, reversed the decision, and reiterated that there was a duty for the doctor to discuss with the patient both the material risks involved in the medically preferred treatment and any alternative treatment options. In the claimant’s case, it was found that the risk of shoulder dystocia was substantial and should have been disclosed. The claimant was entitled to consider this risk against the risk to both mother and baby of a caesarean section. It was not in dispute that had the baby been delivered by caesarean section it would have been unharmed. The Supreme Court found that had the risks been discussed, the claimant would have elected to have a caesarean. The Supreme Court finally concluded that “an adult person of sound mind is entitled to decide which, if any, of the available forms of treatment to undergo. The doctor is therefore under a duty to take reasonable care to ensure that the patient is aware of any material risks involved in any recommended treatment and of any reasonable alternative or variant treatments”.

The importance of the decision can be shown by the fact that it has already been applied to a series of cases and influenced the outcome of several other cases. This will be of huge importance in the future to claimants bringing clinical negligence cases on the issue of consent. All of the above refers to law in the UK; however, the concept behind these decisions may theoretically apply to any jurisdictions around the world. Our patients are the only ones who have the rights of making decisions about what they consider being the best treatment or care options for them. This may sometimes conflict with the clinician’s preference of best medical treatment, but in the final analysis, the decisions regarding care must rest with the patient.

In order to avoid the risk of lawsuits for medical negligence, our duty should be simply to provide all possible information to help patients make decisions and to respect their final decisions once made (http://www.gmc-uk.org/guidance/ethical_guidance/consent_guidance_index.asp.). The problem then is not whether or not we should provide patients with information about their treatment options for treating SUI, but it becomes an issue of what is enough information and how do we best present and document that provides that information. Thankfully, long gone are the days when no questions were asked of the medical profession, as now a patient’s autonomy in the decisionmaking process must be considered above all else.

### Authors’ opinion and advice

We strongly believe that the exchange of information between doctor and patient is central to good decision making. The relationship between clinician and patient should be a partnership based on openness, trust, and good communication. Therefore, we as clinicians should use our experience and knowledge to explain the options to the patient, including the option to forgo any treatment, setting out the potential benefits, risks, burdens, and side effects of each treatment option without overwhelming the patient. While the doctor may recommend a particular option, the patient must weigh up the potential benefits and risks and decide which option he/she wishes to pursue. Therefore, it is mandatory that clear, accurate information about the risks of any proposed investigation or treatment are presented in such a way that patients can understand and can help them reach an informed decision. Only with risks explained and understood can a patient determine what treatment path is likely to achieve their desired outcome against what is an acceptable risk. Risk should be considered as any side effects, complication, or failure of an intervention to achieve the desired aim. Since different risks will have different levels of importance to different patients, it is mandatory to inform our patients of any potential risk, even if the likelihood is very small (http://www.gmc-uk.org/guidance/ethical_guidance/consent_guidance_index.asp.).

### Use of checklists

In light of the discussion so far, what we should do and what will happen next remains uncertain. There are suggestions from several scientific societies and even national healthcare institutions that patients be given some type of checklist to supplement informed consent documents, when surgery for SUI with or without mesh is performed, as a means of proving that we have discussed the multitude of rare complications associated with surgery. So, our question is: Is this reasonable, or have we given in to societal pressures to go beyond what is reasonable?

### Checklists may be the answer

Can the use of a supplementary checklist promote full disclosure about midurethral slings and protect both patients and clinicians? Checklists are well established as a safety tool worldwide, and while they may seem mundane, they are a critical step in protecting against human limitations to preserve/protect safety. They work well in high-risk settings, such as air transport, nuclear power industry, weapons systems, space flight, and operating rooms. World Health Organization (WHO) surgical checklists have demonstrated the ability to significantly reduce common complications in the pre-, intra- and postoperative arenas. Therefore, if a simple checklist could play a key role in ensuring that the patient understood all benefits, risks, and alternatives to the procedure in question and is aware of the lack of published data in case of further pregnancy despite the fact that a vaginal delivery after continence surgery does not seem to be contraindicated, then it may be sensible to start using them in the routine informed consent process. In this sense, its role would be to protect both the patient and the doctor. What better record of a discussion can there be?

However, preoperative checklists, unlike safety checklists, are not an end unto themselves. In other words, having a signed informed consent checklist is not the same as having a safety checklist signed off. The typical safety checklist is a simple series of steps that have objectively defined understanding, for instance: “Was the patient identified by name, birth date, and medical record number?” Informed consent checklists are much more subjective and require the patient to understand complex relationships between risk and benefit. So simply having a patient initial a checklist item without their understanding does not provide any benefit to the patient. Therefore, the informed consent checklist should only be an aid to the informed consent process, not unlike any other type of educational patient handout that describes a procedure. We should remember to continue with an informed consent process that covers the major issues and medical notes that record the information provided at the preoperative consultation regarding the procedure being offered, alternatives, expected outcomes, and potential complications. Using a checklist as a means of furthering discussions is appropriate but cannot take the place of the informed consent process.

We have never employed a procedure-specific checklist justifying other gynecologic surgeries, such as a vaginal hysterectomy or tubal ligation. However, we are living in a different and more demanding society that has led us to alter how we approach the informed consent process, particularly in regard to polypropylene mesh in vaginal surgery. This will require spending more time during the surgical consent process to be certain that all risks, options, and outcomes are covered and patients understood the information provided. It could be argued that one downside to employing an informed consent checklist is that we appear to be defensive and trying to justify performing the current gold standard procedure for correcting SUI. Instead of using it as a means of trying to avoid/reduce the risk of lawsuits, we should use it to promote an open discussion with our patients about risks and benefits of the midurethral sling.

Finally, we provide the following suggestions as to how we can continue in the current situation:

First, clinicians should continue to have an open and frank informed consent discussion with their patients. We must be sure that clinicians around the world routinely use patient handouts that describe how midurethral slings work, their risks and benefits, and the possible alternatives. If a clinician decides to use a checklist, it should be done under the guise of promoting further patient discussions and not as a defensive strategy to avoid potential litigation. If patients question the use of a checklist, we should have a frank and open discussion about how outside medicolegal pressures are forcing our hand, but that it is only a means to ensure that we have discussed all current concerns that outside sources are introducing into our discussion.

In addition, there should be a public awareness campaign about how midurethral slings are considered the gold standard, as they have fewer complications and similar efficacy with better long-term results than any other continence procedure currently available.

Finally, as recommended by the FDA [1, 2], National Institute for Health and Care Excellence (NICE) (https://www.nice.org.uk/guidance/cg171), and more recently supported by Science Committee on Emerging and Newly Identified Health Risks (SCENIHR) [7], we should ensure that only experienced and skilled surgeons implant permanent midurethral slings and manage complications related to them. Physicians should be considered as competent if they manage to demonstrate that:

1. They have received appropriate training in the management of SUI and associated disorders.
2. They work within a multidisciplinary team.
3. They regularly carry out antiincontinence surgery in women, with sufficient workload per year to maintain a high skill level;
4. Their training, experience, and practice equates to the standards laid out for newly trained surgeons.

Ideally, women should be able to access public records, audit data, and registries of national and international professional scientific societies to better understand the published success, failure, improvement, and complication rates associated with midurethral slings. Information leaflets covering the background, alternative treatments, and issues about the surgery should be given to and discussed and reviewed with the woman prior to surgery.

## Conclusions

The current medicolegal environment surrounding midurethral slings has caused us to rethink some of our informed consent processes. We should use this moment as an opportunity to promote better understanding by our patients of available treatment options and allow them to weigh the risk–benefit equation for their choices in all forms of treatment we offer. Using an informed consent checklist should not be confused with having a frank and open discussion with patients. A checklist should only serve as a starting point in the informed consent process and not be a defensive procedure to reduce the risk of litigation against a surgeon. Therefore, the use or not of a checklist becomes a personal decision by a given clinician, but either way, that clinician should be competent in inserting midurethral slings and openly discuss their risks and benefits with the patient.
